# EMBRACE-WATERS statement: Recommendations for reporting of studies on antimicrobial resistance in wastewater and related aquatic environments

**DOI:** 10.1016/j.onehlt.2021.100339

**Published:** 2021-10-19

**Authors:** Nasreen Hassoun-Kheir, Yoav Stabholz, Jan-Ulrich Kreft, Roberto de la Cruz, Arnaud Dechesne, Barth F. Smets, Jesús L. Romalde, Alberto Lema, Sabela Balboa, Carlos García-Riestra, Eva Torres-Sangiao, Ami Neuberger, David Graham, Marcos Quintela-Baluja, Dov J. Stekel, Jay Graham, Amy Pruden, Joseph Nesme, Søren Johannes Sørensen, Rupert Hough, Mical Paul

**Affiliations:** a Infectious Diseases Institute, Rambam Health Care Campus, HaAliya HaShniya St 8, Haifa 3109601, Israel; b The Ruth and Bruce Rappaport Faculty of Medicine, Technion – Israel Institute of Technology, Efron St 1, Haifa 3109601, Israel; c School of Biosciences, Institute of Microbiology and Infection (IMI), Centre for Computational Biology (CCB), University of Birmingham, Birmingham, UK; d Technical University of Denmark, Department of Environmental Engineering, bygning 115, Bygningstorvet, 2800 Kongens Lyngby, Denmark; e Department of Microbiology and Parasitology, CIBUS-Faculty of Biology, Universidade de Santiago de Compostela, Santiago de Compostela 15782, Spain; f CRETUS, Universidade de Santiago de Compostela, Santiago de Compostela 15782, Spain; g Department of Microbiology and Parasitology, University Hospital Complex of Santiago (CHUS), Spain; h Escherichia coli Group, Research Foundation Institute (FIDIS), University Hospital Complex (CHUS), Santiago de Compostela, ES, Spain; i School of Engineering, Newcastle University, Newcastle upon Tyne, UK; j School of Biosciences, University of Nottingham, Sutton Bonington Campus, College Road, Loughborough LE12 5RD, UK; k University of California, Berkeley School of Public Health, Berkeley, CA, USA; l The Charles Edward Via, Jr. Department of Civil and Environmental Engineering, Virginia Tech, Blacksburg, VA, USA; m Section of Microbiology, Department of Biology, Faculty of Science, University of Copenhagen, 2100 Copenhagen, Denmark; n Information and Computational Sciences, The James Hutton Institute, Aberdeen AB15 8QH, Scotland, UK

**Keywords:** Antimicrobial resistance, Reporting, Recommendations, Aquatic, Environment, One health

## Abstract

**Background:**

A One Health approach requires integrative research to elucidate antimicrobial resistance (AMR) in the environment and the risks it poses to human health. Research on this topic involves experts from diverse backgrounds and professions. Shortcomings exist in terms of consistent, complete, and transparent reporting in many environmental studies. Standardized reporting will improve the quality of scientific papers, enable meta-analyses and enhance the communication among different experts. In this study, we aimed to generate a consensus of reporting standards for AMR research in wastewater and related aquatic environments.

**Methods:**

Based on a risk of bias assessment of the literature in a systematic review, we proposed a set of study quality indicators. We then used a multistep modified Delphi consensus to develop the EMBRACE-WATERS statement (rEporting antiMicroBial ResistAnCE in WATERS), a checklist of recommendations for reporting in studies of AMR in wastewater and related aquatic environments.

**Findings:**

Consensus was achieved among a multidisciplinary panel of twenty-one experts in three steps. The developed EMBRACE-WATERS statement incorporates 21 items. Each item contains essential elements of high-quality reporting and is followed by an explanation of their rationale and a reporting-example. The EMBRACE-WATERS statement is primarily intended to be used by investigators to ensure transparent and comprehensive reporting of their studies. It can also guide peer-reviewers and editors in evaluation of manuscripts on AMR in the aquatic environment. This statement is not intended to be used to guide investigators on the methodology of their research.

**Interpretation:**

We are hopeful that this statement will improve the reporting quality of future studies of AMR in wastewater and related aquatic environments. Its uptake would generate a common language to be used among researchers from different disciplines, thus advancing the One Health approach towards understanding AMR spread across aquatic environments. Similar initiatives are needed in other areas of One Health research.

## Highlights

• One Health research links experts from different disciplines assessing antimicrobial resistance.
• Non transparent reporting in environmental studies hinders data communication.
• Transparent reporting ensures research reproducibility and generalizability.
• EMBRACE-WATERS lists recommendations for reporting of studies on AMR in aquatic environment.
• EMBRACE-WATERS can assist authors in improving reporting quality of studies.

## 1 Background

Antibiotic resistance genes (ARGs) and antibiotic resistant bacteria (ARB) are released into and distributed across all aquatic environmental compartments [1]. ARGs can be found in municipal and healthcare wastewater, wastewater treatment plants (WWTPs), surface water, groundwater and even in drinking-water [2]. Abundance of AMR bacteria in recreational areas, drinking water, ambient air, and food sources such as shellfish suggests risk for human exposure; but the effect of this exposure is poorly quantified [3]. Environmental spread of AMR residues can also disseminate through the food chain, with significant public hazard [4]. Water-based epidemiology of infectious agents can assist in monitoring trends of infectious outbreaks on a-community scale [5]. For example, estimated viral RNA copy numbers of SARS-CoV2 from Australian wastewaters were used to estimate the number of infected individuals with COVID-19 in the same areas [6]. Water-based epidemiology of AMR can improve surveillance of AMR in human clinical pathogens in an affordable and acceptable manner [7].

Research on the environmental epidemiology of AMR in water compartments and its determinants is integral to a One Health understanding of the problem of AMR [8]. The World Health Organization developed a global action plan in 2015 to tackle AMR; among its primary objectives was to improve the understanding of how AMR develops and spreads, including between humans, animals, food and water, and across the environment [9]. One of the key components of this plan called to support collaborative research, working across human medicine, veterinary medicine, public-health, and environmental scientists within a “One Health Initiative” [10,11].

As One Health research involves experts from different backgrounds and professions, standardized reporting is vital to improve communication among disciplines. Clarity and consistency of research papers is crucial for the generation of novel One Health evidence. A common language is needed to make the environmental literature more accessible to clinicians who deal with AMR in healthcare settings. Inappropriate reporting might involve under-reporting of crucial aspects in study's context, methods and mainly results. Selective reporting or analysis occurs when only some findings are reported, depending on the nature and direction of the results (often excluding statistically insignificant results), and can introduce bias [12,13,14]. Missing descriptions of study methods might mask both strengths and limitations of a study and is a major obstacle to reproducibility [15], which is an increasingly recognized quality criterion in research [16,17]. Reproducibility and replicability, denote the ability to use the same methods to re-perform a scientific work in the same experimental system or in a different one and obtain consistent results, respectively. Robustness and generalizability measure the ability to use different methods to re-perform a scientific work in the same experimental system or in a different one and obtain consistent results, respectively [18]. Hence, incomplete or inadequate reporting might adversely affect all aspects of study validity.

We aimed to generate a consensus reporting standard for AMR research in wastewater and related aquatic environments, resulting in the EMBRACE-WATERS (rEporting antiMicroBial ResistAnCE in WATERS) statement. We hope that implementation of these recommendations will improve the comprehensibility, transparency, comparability and reproducibility of such studies by all communities working under the One Health umbrella.

## 2 Methods

**Fig. 1:**
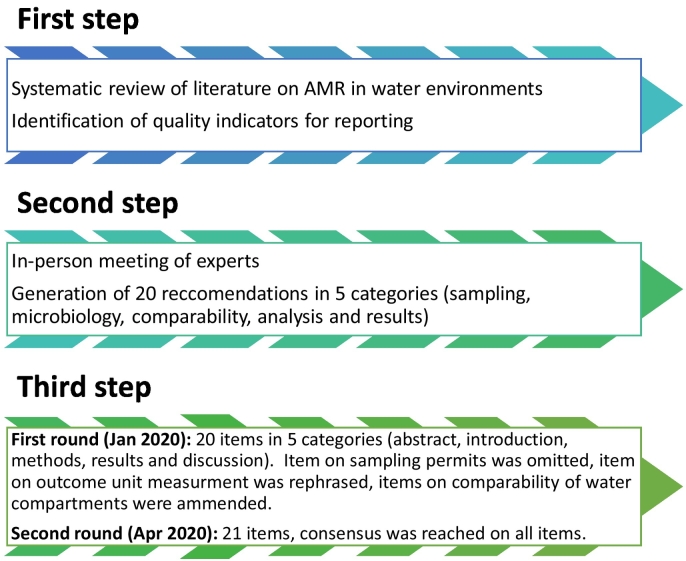
Work flow scheme.

The consensus process comprised of a three-step modified Delphi method [19] (Fig. 1).

### 2.1 Generating reporting standards

In the first step, we identified quality indicators for reporting through a systematic review of the literature on AMR in urban water cycles [20]. The second step included a face-to-face meeting of the multidisciplinary panel of experts (JPI-EC-AMR project DARWIN – Dynamics of Antimicrobial Resistance in the Urban Water Cycle in Europe group authors) in October 2019 to discuss the indicators. This panel included experts in microbiology, environmental engineering, water experts, modeling and computational biology, clinical infectious diseases, infection control and other fields (supplement S1). Based on discussions in this meeting, potential reporting standards for studies on AMR in aquatic compartments were defined.

### 2.2 Reaching consensus (as many rounds as required)

In the next step, the expert panel was expanded and questionnaire rounds were used to solicit feedback and reach consensus on the suggested reporting standards [21]. A draft of potential reporting standards was sent to experts including all panel participants via SurveyMonkey®. In each round, the panel members were asked to anonymously rate the items on the basis of their knowledge and experience with respect to relevance, importance and feasibility, using a 5-point Likert scale (1 = strongly disagree to 5 = strongly agree) [22]. In the first round, the experts could also suggest additional items not included in the list. In each round, they could provide suggestions for rephrasing the existing items. After each round, the list was revised based on the responses. We performed sequential rounds until consensus was reached on all items [23].

Median scores and interquartile ranges (IQRs) were calculated for responses to each statement in each round to assess agreement and level of consensus, respectively. Responses with a median ≥ 4 and with IQR <2 (agreement and consensus) were carried to the next round; statements with a median ≥ 3 with IQR ≤2 (intermediate agreement/consensus) were revised for the subsequent round; and items with a median < 3 or IQR >2 were excluded. Descriptions were accepted if 80% of the experts agreed on their content [24]. Irrespective of the scoring, items were labeled for revision if more than three experts made similar comments regarding the phrasing.

## 3 Results

### 3.1 Generating consensus on reporting recommendations

During the DARWIN expert group meeting held on Oct 2019, the risk of bias scoring results of the systematic review were presented [20]. Based on discussion and feedback, we generated a list of 20 preliminary reporting recommendations in five categories: sampling, microbiology, comparability, analysis and results (Fig. 1).

After the meeting, this list of reporting recommendations was released, and the first Delphi round was launched on Jan 13th, 2020. Among 21 experts (18 from the DARWIN group and three outside DARWIN), one recommendation statement was rejected (“provide permits needed for sampling”). Intermediate level of consensus was reached on a second item (“preferably report outcomes as concentration units (CFU/volume or gene copies/volume) rather than proportion of resistant bacteria or absolute numbers”), which was rephrased. Based on the experts' comments, two items on comparability between water compartments were merged with other items.

The second round was launched on April 28th, 2020 and included 21 items. Among 21 experts, agreement was reached on all items. Suggestions for rewording without change in meaning were accepted and adopted.

### 3.2 EMBRACE-WATERS checklist

The EMBRACE-WATERS checklist includes 21 recommendations for reporting in future studies of AMR in wastewater and related aquatic environments (Table 1). These items relate to the article's title and abstract (item 1,2), introduction (item 3), methods (items 4–14), results (items 15–19) and discussion (items 21–21). Below, we present an explanation for the recommendations to provide the rationale underlying each item. We provide examples from published studies to illustrate its expected use by authors. Wording was copied from the original papers in the example quotations.

**Table 1:** EMBRACE-WATERS checklist – recommendations for reporting on AMR in wastewater and related aquatic environments.

Section/topic	Number	Checklist item
Title and abstract		
Title	1	Describe the environmental compartment and antimicrobial resistance studied
Abstract	2	Provide a structured summary including implications of key findings

Introduction		
Background	3	Describe the scientific background and the rationale of the study

Methods		
Planned location	4	Report on predefined sampling locations
Sample types	5	Describe sample types in each location
Technique	6	Describe the sampling techniques
Equipment	7	Describe the type of equipment used for sampling
Sample volume	8	Report the volume of the samples from all locations for each analysis
Sample processing	9	Report sample processing by sample type and on-site preservation methods
Source characterization	10	If sampling water from a point source (agricultural water, raw sewage inlet, WWTP effluent, etc.) or downstream the point source, report the exact source and its characteristics; In the absence of a point source, report characteristics of the watershed.
Microbiological methods	11a	Describe the microbiological methods used to detect bacteria
	11b	Report how antibiotic resistance was assessed
Analysis plan	12	Describe the data analysis or analytical pipeline planned for comparison. Report on use of statistical tests
Sample size	13	Calculate the number of samples required to address the research question (statistical power calculation)

Results		
Locations	14	Report and describe actual sampling locations
Dates and weather	15a	Report the season, dates and frequency of sampling
	15b	Provide description of weather conditions in the period leading up to the sampling, precipitation and any other external factors
Water quality indicators and metadata	16	Report general water quality conditions and any other meta-data evaluated
Results	17	Report results per location, including negative results
Units of analysis	18	If possible, report outcomes as concentration units (and normalized concentration) and provide confidence intervals for all results
Post hoc analysis	19	Describe the actual statistical analysis performed if different from the planned analysis, report on additional post-hoc analyses if done

Discussion		
Interpretation	20	Discuss the study findings in the context of existing evidence
Limitations	21	Address the study limitations

#### 3.2.1 Title and abstract

*Item 1: “Title - Describe the environmental compartment and antimicrobial resistance studied”.*

**Example:** “Multidrug-Resistant and Extended Spectrum Beta-Lactamase-Producing *E. coli* in Dutch Surface Water and Wastewater” [25].

**Explanation:** The title should let readers easily identify the tackled antimicrobial resistance trait and the environmental setting of the study. It should be concise, clear and informative [26]. *In the example,* the resistant bacteria, the resistance mechanism and the sampled environment are clearly stated.

*Item 2: “Abstract - Provide a structured summary including implications of key findings”.*

**Example:** “Among simple surrogates, dissolved oxygen (DO) most strongly correlated (inversely) with total AR gene concentrations (Spearman's ρ 0.81, P < 0.05). We suspect this results from minimally treated sewage inputs, which also contain AR bacteria and genes, depleting DO in the most impacted reaches. Thus, although DO is not a measure of AR, lower DO levels reflect wastewater inputs, flagging possible AR hot spots […. We] propose combining DO data and prospective modeling to guide local interventions, especially in LMIC rivers with limited data” [27].

**Explanation:** Readers often read only the abstract or use the abstract to decide whether to access the full-text. Thus, essential information should be clearly presented [28]. The abstract should provide the reader with the study aims, methods of sampling and microbiological analyses, main findings and conclusions or implications. Absolute numbers should be presented for all results, and confidence intervals or other dispersion measures should be provided. When reporting percentages, the nominators and denominators should be reported. By informative reporting in abstracts, readers should be able to easily examine the study relevance to their setting. Information not provided in the full-text should not be presented in the abstract. Specific headings should be adapted to the journal-specific requirements. Abstracts without headings can follow this structure implicitly. *In the example,* we quoted the implications of key study findings, which are presented in a coherent and simple manner. In the referenced abstract, the journal requested an abstract without headings; however, the abstract is internally structured into background, aim, methods, results and implications.

#### 3.2.2 Introduction

*Item 3: “Background - Describe the scientific background and the rationale of the study”.*

**Examples:** (1) “[…] It has been documented in different studies that members of the genus *Aeromonas* can harbor genes encoding beta-lactam and plasmid mediated quinolone resistance. [… The] contribution of hospital effluents for the dissemination of these bacteria […] remains unclear. Thus, the aim [… was] to assess the role of *Aeromonas* spp. on the dissemination of antibiotic resistance, […] to elucidate if the hospital effluent could be considered its major reservoir or if, in contrast, *Aeromonas* spp. could be indicators of antibiotic resistance from non-clinical sources […]” [29].

(2) “This study is globally relevant because India is the largest consumer of antibiotics for personal use in the world and β-lactams are among the most commonly used antibiotics. […] We suspect antibiotic misuse in places like India partially explains the early evolution of CRE strains, including blaNDM-1 positive isolates. Therefore, although New Delhi presents an extreme case, it is a template for understanding AMR spread in any large city without adequate wastewater management; a common scenario in the developing and emerging world” [30].

**Explanation:** The background should provide information directly pertinent to the study question and justify the study in light of existing knowledge. Avoid opening with well-known, broad-sweeping, overgeneralized and oversimplified statements, such as overuse, misuse, and abuse of antibiotics, antibiotics are the root cause of the spread of AMR, etc. Instead, be focused, provide information and actual statistics related to the tackled AMR research question in the paper [31].

Define the rationale of your study and your hypotheses. Provide an overview of the evidence related to the study hypothesis, the knowledge gaps, and place the study in the epidemiological context (resistance prevalence) of the study location. Preferably, relate the main study aims to some implication on human or animal health. *In the first example,* the authors describe the state of knowledge and gaps they intend to explore. *In the second example*, the authors rationalize their planned study location.

#### 3.2.3 Methods

*Item 4: “Planned location – Report on predefined sampling location”.*

**Example:** “Sampling across the New Delhi wastewater network included hospital effluents, open and sub-surface sewer drains, STPs and final receiving waters. The network comprised 20 drain sites, 12 hospital waste outfalls, effluents from 12 STPs, and five sites along the Yamuna River, which is the ultimate receptacle for wastewaters from the city” [30].

**Explanation:** Planned sampling locations should be defined with the explicit rationale for their selection. Locations can be decided upon considering different factors, e.g., previous findings from the same area, proximity to certain places and regulatory issues. *In the example,* the locations are explicitly provided with a justification for sampling from the Yamuna River.

*Item 5: “Sample types – Describe sample types in each location”.*

**Example:** “Samples from raw (untreated) urban wastewater (UW) […] were collected during 24h from the influent wastewater by a sampling device at the treatment plant that allowed continuous sampling. Another three samples were collected in the same manner from the treated urban wastewater (TW) at the exit to the Baltic Sea. Six samples from hospital wastewater (HW) were collected […] by lowering a 50mL flask every 10–15 min during 4h into the wastewater flow and pooling the aliquots” [32].

**Explanation:** AMR epidemiology is dependent on location and setting, thus the precise location of the empirical study is critical to interpretation of the results. Sampled materials and the exact aquatic environment in each location should be reported. This includes, but is not limited to: surface water, sediments, sewage, sludge, treated or raw wastewater. *In the example,* different materials were sampled in different locations, each is defined and described separately.

*Item 6: “Technique – Describe the sampling techniques”.*

**Example:** (1) “Flow-proportional sampling (over 24 hours) was used for sampling hospital wastewater and WWTP influent and effluent. […] For the surface water samples, grab samples (5 L) were taken at 50 cm downstream of the two effluent pipes… at a depth of 20 cm, in order to obtain a river sample under the direct influence of WWTP effluent” [33].

(2) “Samples were measured in situ using hand-held probes (Mettler Toledo™, FG3 FiveGo™,

and Jenway Model 350 pH Meter) to characterize wastewater conditions, temperature, pH, dissolved oxygen, and conductivity” [34].

**Explanation:** A full description of the techniques applied allows appraising the quality of the study and should ensure the study's reproducibility. Sampling techniques can include grab samples, composite samples, proportional sampling, etc. In addition, the use of manual and/or automatic samplers should be described. *As in the first example,* if different techniques are used in different locations, each should be clarified. The reporting could be improved by providing information on the distance between the two effluent pipes and the discharge volume relative to river flow, i.e., the extent of dilution. *In the second example*, the authors describe the techniques used for characterizing wastewater conditions and meta-data.

*Item 7: “Equipment – Describe the type of equipment used for sampling”.*

**Example:** “samples were collected along the pathway from medical center effluents to a river impacted by a WWTP using autosamplers (ISCO 6700s, Roucaire, Courtaboeuf, France)” [35].

**Explanation:** The type of equipment used is important to interpret the quality of the study and allow replicability. Preferably include manufacturers and model numbers in the manuscript or supplementary material.

*Item 8: “Sample volume – Report the volume of the samples from all locations for each analysis”.*

**Example:** “A volume of 1 L of wastewater and 10 – 20 L of surface water was collected” [36].

**Explanation:** Reporting of the volume sampled allows comparability between locations within a study and between different studies. Preferably, report why these volumes were chosen (e.g., detection limits of key methods, different preservation methods for each method)”. Different volumes sampled in different locations should be justified.

*Item 9: “Sample processing – Report sample processing by sample type and on-site preservation methods”.*

**Example:** “Samples were transported to the laboratory in portable coolers and were stored at 4°C until treatment. All samples were processed directly or within a maximum of 20 h” [37].

**Explanation:** Onsite sample processing and preservation methods applied to the sample from sampling until lab processing can affect the study results. This also includes time periods from sampling until field or laboratory processing, or between field and laboratory processing. *In the example*, mode of preservation and time until laboratory processing are described.

*Item 10: If sampling water from a point source (agricultural water, raw sewage inlet, WWTP effluent,* etc.*) or downstream the point source, report the exact source and its characteristics; In the absence of a point source, report characteristics of the watershed.*

**Example:** “Untreated effluents were collected from WWTP with the capacity of 2.64x10^4^ m^3^ per day and the hydraulic retention time (HRT) equaling 24h. The treatment process involves preliminary treatment (screening and grit removal), primary treatment (gravity sedimentation tanks) and secondary treatment (activated sludge with deep aeration using a Passavant rotor) followed by secondary sedimentation” [38].

**Explanation:** Characteristics of the point source sampled are crucial to the study results. For example, for a WWTP, report on the size, functionality, operating conditions and treatment processes applied. *In the example,* the WWTP capacity and the stages of the wastewater treatment processes are reported. In the absence of point sources, report characteristics of the watershed from which rainfall runs off into the water body, such as land cover, land use and topography.

*Item 11: “Microbiological methods: (a) Describe the microbiological methods used to detect bacteria (b) Report how antibiotic resistance was assessed”.*

**Example:** “1 mL of well-homogenized […] sewage/water was diluted in 9 mL sterile saline solution (0.85% NaCl) […]. Subsequently, 0.1 mL of each suspension was placed on CCA ES medium and incubated for 24 h at 35±2 °C. […] Disks containing cefotaxime (CTX–5 mg), ceftazidime (CAZ–10 mg) […] were placed on inoculated Mueller–Hinton agar plates. After overnight incubation at 37 °C, resistance was estimated […] EUCAST (2012). Identification for ESBL production […] was confirmed by […]. Genomic DNA was extracted from 107 selected ESBL-positive […] *E. coli* […]. The regions of the blaCTX-M group 1, blaCTX-M group 9, blaSHV, blaTEM and blaOXA genes […] were amplified by the PCR method […]” [38]*.*

**Explanation:** The microbiological methods used, whether genotypic or phenotypic, and the quality of their application affect the study's findings. The agents (e.g. bacteria, genes, plasmids, etc.) and processes (e.g. growth, gene transfer, plasmid conjugation, etc.) studied should be reported. Microbe detection, identification and enumeration can include culture-dependent or culture independent methods, as relevant, and should be detailed. The microbiological methods used to detect or measure resistance can include phenotypic characterization (culturing information including type of agar, antibiotic concentrations used in media or disks, and growth conditions), quantitative and qualitative PCR and next generation sequencing (report DNA extraction methods, planned genomic analysis and metagenomics as relevant). Furthermore, if novel methods have been used, they should be validated against established reference methods and sufficient detail provided to enable reproducibility. If there are no gold standard methods available, such as in the rapidly developing field of metagenomics, it is advisable to use an ensemble of methods to ensure the results are robust and not too dependent on the particular method chosen [39,40].

*Item 12: “Analysis plan – Describe the data analysis or analytical pipeline planned for comparison. Report on use of statistical tests”.*

**Examples:** (1) “Two-sample t-test was used to determine the differences between ampicillin, streptomycin, tetracycline, sulphonamide and ciprofloxacin resistant *E. coli* after treatment in WWTP and between the effluents of WWTP” [41].

(2) “A log(X+1) transformation was applied to datasets and a resemblance matrix was calculated by Bray–Curtis analysis. Clustering patterns were statistically validated by an Analysis of Similarity (ANOSIM) procedure using 999 iterations to test the significance of the clustered groups. A SIMPER analysis was used to determine the similarities in microbial community composition between samples” [36].

**Explanation:** Complete description of the analysis is required to understand the study and allow replicability. Preplanned analysis plans are important to avoid selection of details reported or their handling (selective reporting). This includes, but is not limited to, any bioinformatics software and statistical tests used; all parameter values, settings and thresholds should be reported. *In the first example,* the planned statistical analysis is explained. *In the second example,* a full report of the analytical process, software and formulas used in a metagenomic research paper are reported.

*Item 13: “Sample size – Calculate the number of samples required to address the research question. Provide statistical power calculation when relevant”.*

**Example**: “Determination of sample size is mainly based on the statistical variance for the population, survey accuracy (relative error), and confidence of probability. The relative allowable error of the study area is less than 10%, and the confidence of probability is greater than 95% [...] the sample size of the survey required 482 survey samples” [42].

**Explanation:** A sample size or power calculation is necessary to determine how many samples are required to answer the study question, or what power a given number of samples has to provide a robust answer. While the methodology for determining the sample size of environmental studies (e.g. number of samples, volume) is not well-established, providing an estimate of the required sample size or some justification for the sample size plan will strengthen the study conclusions. *In the example*, a sample size calculation was performed for surveys of groundwater resource nitrate content evaluation.

#### 3.2.4 Results

*Item 14: “Locations - Report and describe actual sampling locations”.*

**Example:** “Wastewater samples were taken in the city of Sneek, The Netherlands (33,855 inhabitants) including the following locations: wastewater from a nursing home (220 beds), a hospital (300 beds), a community wastewater collection point (80 households), and the influent and effluent of the corresponding municipal WWTP […]. Surface water samples were collected from the receiving surface water of the Geeuw canal at two locations, 330 m south-west (N 53_02015.10”, E 5_63072.76″) and 388 m north-east (N 53_02072.15″, E 5_64028.97″) from the WWTP discharge point (N 53_02038.85″,E 5_64003.20″)” [43].

**Explanation:** The actual sampling locations might be different from the planned study location. If so and in any case, the actual locations in which the sampling was done should be reported in detail. Use of descriptive maps, illustrative sampling plans and GPS coordinates is encouraged. The area of sampling should be described as urban, semi-rural or rural when relevant. It should be made clear what sources could be contributing to the specific sampling location (e.g., large-scale food-animal production, migratory birds, onsite sanitation systems and networked sewerage). *In the example,* the different locations were described including GPS coordinates for surface water sampling are provided.

*Item 15: “Dates and weather – (a) Report the season and dates and frequency of sampling. (b) Provide description of weather conditions in the period leading up to the sampling, precipitation and any other external factors”.*

**Examples:** (1) “Samples were taken during a period of 2.5 weeks in Spring on four days (Monday 31 March 2014 = t1; Wednesday 2 April 2014 = t2; Monday 7 April 2014 = t3 and Monday 14 April 2014 = t4). Cumulative precipitation in the three days preceding each sampling date amounted to maximally 15 mm. The daily flows amount to 74800 ± 5900 m^3^ for the urban WWTP, and 3390 ± 380 m^3^ for the suburban WWTP during the four sampling days” [33].

(2) “The [river] flow rate during the sampling was estimated at 0.2-0.3m^3^/s, which was about half the WWTP flowrate during the sampling period […]. Such dilution is common in southern Europe in the summer. […] This network provides data relevant to any location with limited wastewater dilution […]. Summer sampling was selected to assess the worst-case scenario in terms of dilution of WWTP effluents in receiving waters” [34].

**Explanation:** Seasonal factors can play a significant role in AMR densities in aquatic environments [44]. In addition, precipitation levels and overflows and changes in water systems can influence study results. The season in which sampling was done and exact dates should be provided. External factors such as weather conditions, precipitation, alterations in wastewater system or other factors that might influence sampling, preceding and during the sampling period should also be described. *In the first example,* the season and exact dates are provided as well as details on the cumulative precipitation preceding the sampling at the different locations. *In the second example,* the rationale of sampling the summer in Southern Europe is explained, to enable the generalization of the study results to other locations with dry climates and limited dilution of wastewater in the receiving rivers.

*Item 16: “Water quality indicators and metadata – Report general water quality conditions and any other meta-data evaluated”.*

**Example**: “Table S-1: Catchment metadata from the different sites. Measured values are from three different weeks per site for pH, conductivity, temperature, [chemical oxygen demand] COD, DNA concentration and bacteria density” [34].

**Explanation:** Water temperature, richness of organic matter and redox conditions can affect the interactions between different chemicals, antibiotics and ARB. Different metals and non-antimicrobial stressors modulate the permissiveness of bacterial communities towards conjugal plasmids and the rates of horizontal gene transfer [45]. Antimicrobials, biocides, heavy metals, disinfectants and non-antibiotic pharmaceuticals can enhance ARGs' transmission [46,47,48]. Water quality conditions and relevant meta-data should be described, including but not limited to: turbidity, pH, water temperature, total and volatile suspended solids, heavy metals, total phosphorus, total nitrogen, dissolved oxygen, chemical and biochemical oxygen demand. *In the example,* Catchment metadata were measured in triplicate per each location and the results are provided in a supplementary table.

*Item 17: “Results – Report results per location, including negative results”.*

**Example:** “Table 1, Antimicrobial resistance among *E. coli* isolated from sewage, river water and air samples” details *E. coli* concentrations and susceptibilities per location (Hospital 1, Hospital 2, Hospital 3, Inflow, Sewage in aeration tank, Outflow, River water, Air near grit chamber, Air near aeration tank and Heterogeneous site) [38]*.*

**Explanation:** Reporting of results from all locations and planned tests is important to avoid selective reporting of significant results only, thus distorting the overall evidence. Report explicitly the results from each sampling-location. Include total number of samples collected in each, and results of microbiological assessment per location. Avoid reporting only aggregate results. The reader should be able to independently interpret results for each location and compare these results. Furthermore, if selecting samples for microbiological analysis (phenotypic or genotypic), the total number of samples and the reason for selection should be provided. *In the example,* the total number of samples and specific results, per location and per resistance determinant of interest, are presented in a table.

*Item 18: “Units of analysis and confidence interval – If possible, report outcomes as concentration units (and normalized concentration) and provide confidence intervals for all results.*

**Examples:** (1) A study assessing the presence of carbapenemase genes provided a table with measured absolute (copies/ml) and relative (copies/16S) abundances of bla_KPC_, bla_NDM_ and bla_OXA-48_ genes in WWTPs, hospital and river waters [49].

(2) A study of β-lactam resistant bacteria and genes in Delhi reported results of all assessed resistance determinants of interest including figures of log copies/ml and 95% confidence intervals in each location in supplementary tables [50].

**Explanation:** Reporting clearly the unit of analysis ensures that research across the world can be compared, understood and replicated [51]. Confidence intervals or other dispersion measures reflect the uncertainty in the study results and the power of the study, while *p*-values do not provide information other than statistical significance [52]. Reporting results using both absolute concentration units (e.g. CFU/volume, gene copies/volume) and normalized concentration units (e.g. gene copies/16S rRNA gene copies) with confidence intervals or another dispersion measures is advised. *In the examples*, concentrations with confidence estimates are provided.

*Item 19: “Post-hoc analysis – Describe the actual statistical analysis performed if different from the planned analysis, report on additional post-hoc analyses if done”.*

**Example:** “Post hoc multi-comparison tests were carried out for sample site, where appropriate […] means were post-hoc adjusted and compared using least square means.” [53]

**Explanation:** Unplanned analyses are weaker than pre-planned analyses, since the former may be driven by interest in the results or statistical significance. Differences between the planned study methods or analyses (as reported in a published or unpublished protocol and as presented in the methods section) and those actually used in the study should be reported. The description should address, including but not limited to, sampling techniques, microbiological methods, and statistical and other data analyses. These differences between plan and actual study, should be justified. *In the example,* post hoc comparisons and analysis methods are transparently presented in the methods section, thus allowing their appropriate appraisal by readers. Defining analyses as post hoc can be declared in the methods or in results sections.

#### 3.2.5 Discussion

*Item 20: “Interpretation – Discuss findings in context of existing evidence”.*

**Example:** “It is most interesting that while we found in our work that the most abundant CRE in sewage in Israel was bla_KPC_ carrying *Klebsiella pneumonia* followed by *Enterobacter cloacae* […] in a previous publication by Xinzhuo Zhang *et al*. bla_KPC-2_ positive *Citrobacter freundii* and *E. cloacae* were the most abundant CRE found in hospital sewage in China. The [diversification] pattern of pan-resistant bacteria in sewage could imply the carrier rate of those organisms in the population” [54].

**Explanation:** The main findings and their added value to the existing evidence should be discussed. Differences and similarities to relevant previous studies in the field should be explained [55]. *In the example,* one of the main findings in the study is stated, and further discussed in light of other studies. A possible interpretation for the difference between both studies is suggested.

*Item 21: “Limitations – Address the study limitations”.*

**Example:** “The main limitation of this study is the lack of quantification of CPE load per sample. This was due to technical limitations [… leading] to a low positive predictive value for detection of a true CPE from the growth obtained on the plate and makes quantitation extremely challenging […]. [T]he study was conducted in a region with very low CPE prevalence and may not be generalizable” [56].

**Explanation:** Rather than leaving the readers to identify and interpret the study limitations, declaring all limitations gives the authors an opportunity to explain whether these have a bearing on results and how. The limitations in study's design, data collection, analyses and results should be described and explained [57]. *In the example,* the authors describe technical limitation faced during the study that made their results not generalizable.

## 4 Comments

A three-step modified-Delphi consensus process was completed among a multidisciplinary panel of experts, to develop a checklist of recommendations for reporting of studies on AMR in wastewater and related aquatic environments. The items included in the EMBRACE-WATERS statement address critical points for reporting and are presented in a structured scientific paper template. The importance of clearly documenting methods, results and analyses is highlighted. Each item is followed by an example from papers on AMR in aquatic environments and an associated explanation.

Reporting of clinical studies is guided by reporting recommendations [58]. Adherence to reporting recommendations was linked, not only to improved reporting quality [59,60], but also to better study designs, more adequately powered studies and enhanced use of standardized methodologies [61]. It also improved the ability to compare among different studies [62]. Recommendations for reporting in environmental research is encouraged [63]; it proved practical and influential in non-clinical research, such as the Overview, Design concepts and Details (ODD) protocol for describing agent-based models [64]. Furthermore, peer reviews based on reporting guidelines improved the manuscript quality, and awareness of the recommendations in an early phase of the study boosted this effect [65].

We suggest a preferred order of item presentation. However, items can be presented differently, depending on the context. For example, the baseline AMR measure in the study settings might be better provided per location in the methods section, if multiple resistance epidemiology settings are included in the study. Previously, the COHERE statement addressed reporting of One-Health studies, but focused on the integration of the human, animal and environmental domains [66]. The present statement provides guidance specifically for reporting of AMR studies in wastewater and related aquatic environments; not for the methods or technical aspects of performing them.

There are recommendations that were not included in our statement. For instance, there is no trial registry database for environmental studies; indeed, none of the studies in our systematic review were registered [20]. Thus, we did not recommend study registration, however, publication of a study protocol will add to the robustness of the research, ensuring lack of selective reporting. To enable full reporting of the study methods and results, the use of supplementary data is encouraged. Another important recommendation, not included in our statement, is making the raw data from scientific studies publicly available. Data accessibility is considered a pillar of scientific development, especially in the era of big data and metagenomics research; but compliance with this call for sharing data is lacking [67]. The format of data shared should be reusable and adapted for digital communications under the FAIR principles [68]. As developments occur, we will update the checklist.

Sample size calculations are not commonly used in aquatic research but are encouraged. We recommend addressing size considerations because biological systems are highly variable and hence sampling designs might have limited capacity to detect differences and quantify changes [69]. Several methods can be used, such as Monte Carlo sampling methods; a quantile methodology to handle outliers and substantial proportions of below-detection-limit observation [70], or power analyses to be used for the detection of significant differences in ARGs or microbial composition in experimental designs [71].

We did not address all types of studies evaluating AMR in aquatic environments, but focused on wastewater and related aquatic environments. Neither did we address studies evaluating risk factors for AMR, studies associating AMR in the aquatic environment with AMR in humans or studies assessing effects of intervention to reduce AMR in aquatic environments. Such studies might need to adhere to further research recommendations that may be found in the EQUATOR network [58]. But all studies that include an assessment of AMR in waters, should as a minimum adhere to the EMBRACE-WATERS reporting recommendations.

## 5 Conclusions

In conclusion, we developed the EMBRACE-WATERS statement through a modified-Delphi consensus process among a multidisciplinary panel of experts. We hope that the present EMBRACE-WATERS checklist will assist both authors and journal reviewers to improve the reporting quality of future studies on AMR in the aquatic environment. We hope that journals will adopt these recommendations. In addition, although not primarily intended for this purpose, it can guide peer reviewers and editors in evaluation of manuscripts in this field. Results from studies following these reporting standards can be aggregated for increased statistical power, used to inform larger scale mathematical models or for discerning regional or temporal trends. We hope EMBRACE-WATERS will also make research on AMR in wastewater and related aquatic environments more relevant to the needs of the medical community in One Health initiatives, and we advocate similar initiatives in other aspects of environmental research with links to human medicine and public health.

## Funding

This study was funded in part by the JPI-EC-AMR project DARWIN (Dynamics of Antimicrobial Resistance in the Urban Water Cycle in Europe), from the Joint Transnational Call for Proposals 2016 “Transnational Research Projects on the Transmission Dynamics of Antibacterial Resistance” grant no 681055 and Newcastle University.

## Author contributions

Conceptualization – all authors, Data curation – NHK, YS, MP; Formal analysis- NHK, MP; Funding acquisition – BFS, DG, Investigation – all authors; Methodology – NHK, MP; Project administration- BFS; Resources – BFS, DG, MP; Software – not relevant; Supervision - MP; Validation - MP; Visualization - not relevant; Writing - original draft – NHK, JK, MP; Writing - review & editing – all authors.

## Declaration of Competing Interest

The authors declare that they have no known competing financial interests or personal relationships that could have appeared to influence the work reported in this paper.

## Appendix A Supplementary data

Supplementary material Image 1
