# Supplementary material for: EMBRACE-WATERS statement: Recommendations for reporting of studies on antimicrobial resistance in wastewater and related aquatic environments
Source: One Health. 2021 Oct 19;13:100339. doi: 10.1016/j.onehlt.2021.100339 (PMC8554267; doi:10.1016/j.onehlt.2021.100339)
Supplement: Supplementary file 1 — Supplementary material [file mmc1.docx]

**Supplement S1: Expert panel members and their expertise**

- **Nasreen Hassoun-Kheir:** Clinical medicine, infectious diseases, infection control & prevention
- **Yoav Stabholz:** Clinical medicine, infectious diseases
- **Jan-Ulrich Kreft:** Mathematical modelling, microbial ecology/environmental microbiology
- **Roberto de la Cruz:** Mathematical modelling
- **Arnaud Dechesne:** Environmental microbiology
- **Barth F. Smets:** Environmental microbiology and environmental engneering
- **Jesús L. Romalde:** Environmental and molecular microbiology, food safety
- **Alberto Lema:** Molecular and computational microbiology and bioinformatics
- **Sabela Balboa:** Molecular biology
- **Carlos García-Riestra:** Clinical microbiology
- **Eva Torres-Sangiao:** Clinical microbiology
- **Ami Neuberger:** Clinical medicine, infectious diseases, antimicrobial resistance
- **David Graham:** water and wastewater engineering & molecular microbiology
- **Marcos Quintela-Baluja:** Molecular biology
- **Dov Stekel:** Computational Biology
- **Jay Graham:** Environmental Health Sciences, specializing in environmental microbiology and infectious diseases
- **Amy Pruden:** Environmental Science & Engineering, specializing in environmental microbiology in wastewater, drinking water, and surface water
- **Joseph Nesme:** Microbial ecology
- **Søren Johannes Sørensen:** Molecular Microbial ecology
- **Rupert Hough:** Environmental risk and exposure modelling/assessment
- **Mical Paul:** Clinical medicine, infectious diseases, antibiotic resistance
